# Supplementary material for: Antioxidant and Anti-Inflammatory Effects of 6,3’,4´- and 7,3´,4´-Trihydroxyflavone on 2D and 3D RAW264.7 Models
Source: Antioxidants (Basel). 2023 Jan 16;12(1):204. doi: 10.3390/antiox12010204 (PMC9855077; doi:10.3390/antiox12010204)
Supplement: Supplementary file 1 [file antioxidants-12-00204-s001.zip › antioxidants-2146090-supplementary.pdf]

Supporting information

Antioxidant and Anti-Inflammatory Effects of 6,3’,4’- and 7,3’,4’-Trihydroxyflavone on 2D and 3D RAW264.7 Models

1. Resources of Chemicals and Reagents.

6,3′,4′-HOFL and 7,3′,4′-HOFL were purchased from INDOFINE Chemical Company, Inc. (USA). Bio-grade DMSO (purity ≥ 99.9%), LPS (from *Escherichia coli*

serotype 055:B5, purity ≥ 99%), c-Src Inhibitor Screening Kit, DCFDA (purity ≥ 97%), bisbenzimide H33342 (purity ≥ 97%) and *t*BHP were purchased from Sigma-Aldrich

Co., Ltd. (Singapore). High glucose DMEM, penicillin-streptomycin antibiotics (1,000 IU/ mL penicillin and 1,000 μg/mL streptomycin), FBS, PBS, BCA Protein Assay Kit,

RIPA lysis buffer and TRIzol reagent were supplied by Thermo Fisher Scientific Co., Ltd. (Singapore). CCK-8 was purchased from Dojindo Molecular Technologies, Inc. (Kumamoto, Japan). Griess reagent and Gotaq® qPCR Master Mix was purchased from Promega Pte Ltd. (Madison, Wisconsin, USA). F-actin staining kit-red fluorescence-Cytopainter was purchased from Abcam (Singapore). Ultralow attachment multi-well

plates were supplied by Corning Inc. (New York, USA). All the antibodies were purchased from Cell Signaling Technology Inc. (Massachusetts, USA). The iScript cDNA synthesis kit, PVDF membranes was supplied by Bio-Rad Laboratories Pte. Ltd. (Singapore). All the primers were purchased from Integrated DNA Technologies Pte. Ltd. (Singapore).

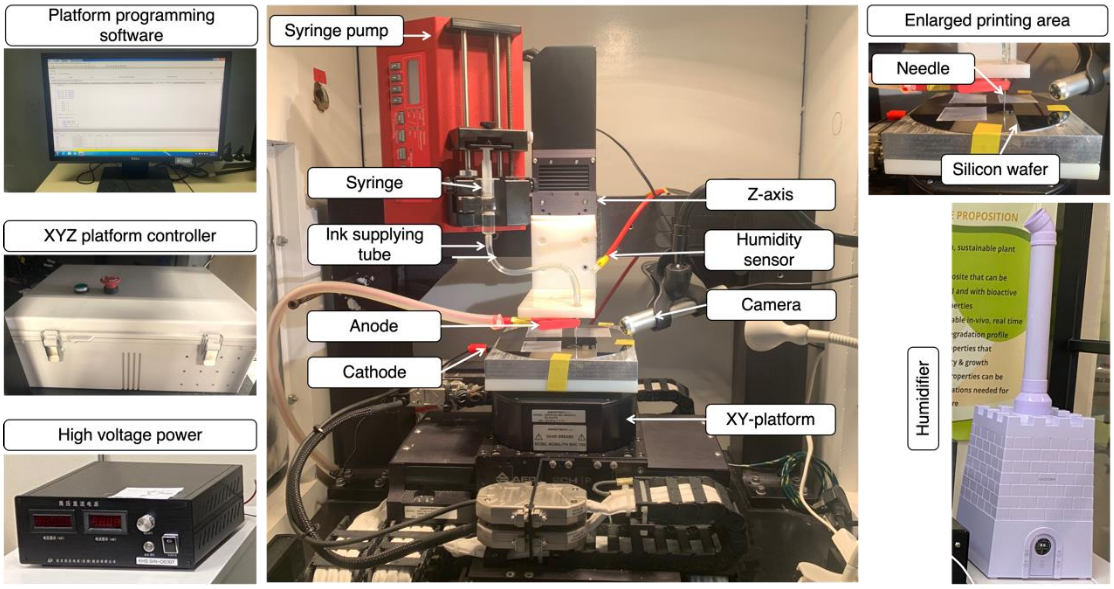

Figure S1 Picture of homebuilt 3D electrohydrodynamic jetting (EHDJ) printer.

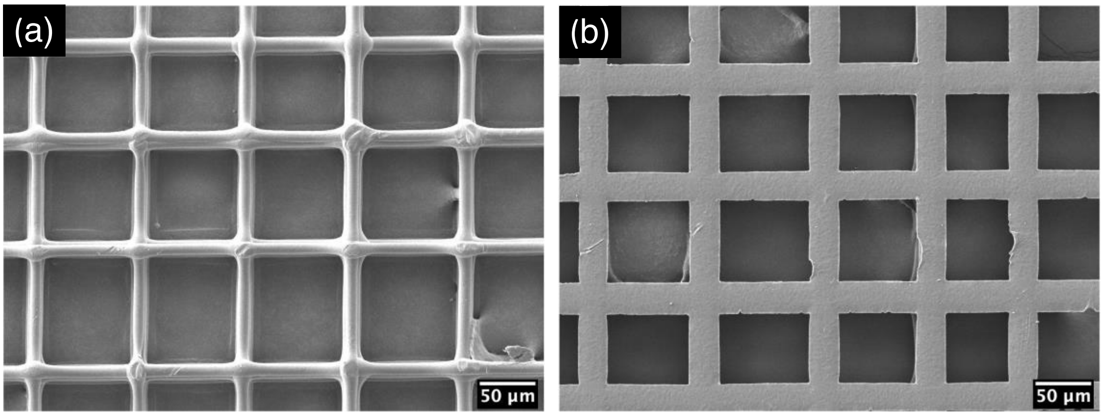

Figure S2 Scaffolds morphology. Top view **(a)** and back view **(b)** of PCL EHDJ-printed scaffolds with 100 μm pore size for 3D cell culturing.

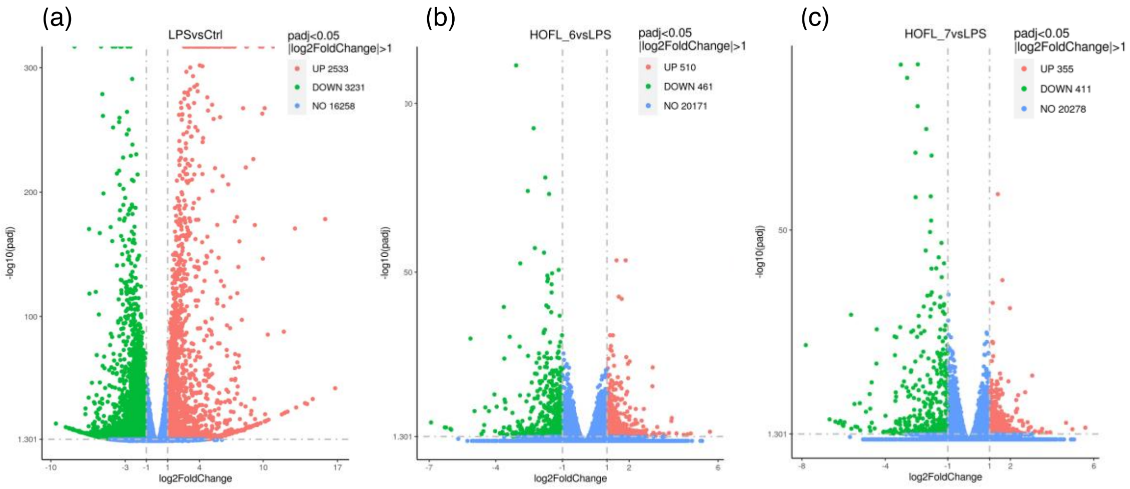

Figure S3 Volcano plots analysis of differential expression genes (DEGs) in RAW 264.7 cells. LPS stimulation led to 2533 genes upregulated and 3231 genes downregulated **(a)**; 6,3´,4´-HOFL treatment led to 461 genes upregulated and 461 genes downregulated in LPS induced inflamed cells **(b)**; 7,3´,4´-HOFL treatment led to 355 genes upregulated and 411 genes downregulated in LPS induced inflamed cells **(c)**. Threshold: padj<0.05, |log2FoldChange|>1.

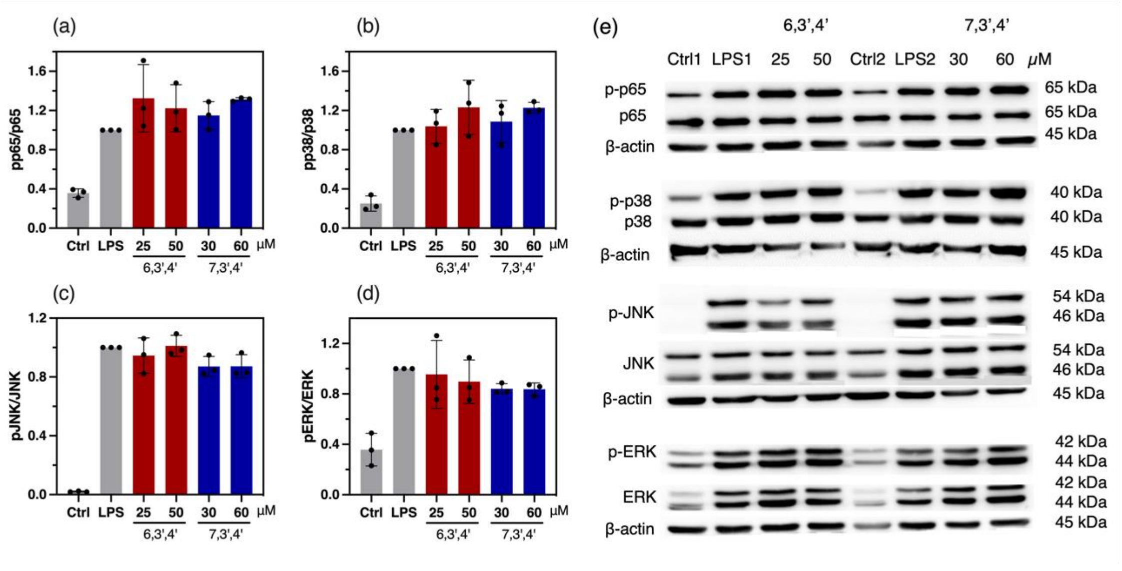

Figure S4 Protein expression of pp65/p65 **(a)**, pp38/p38 **(b)**, pJNK/JNK **(c)**, pERK/ERK **(d)**, and their bands **(e)**. Data are shown as mean ± SD, n = 3. Each result was triplicated. LPS led to the phosphorylation of p65, p38, ERK and JNK, and pretreatment of 6,3´,4´- and 7,3´,4´-HOFL showed no effect on their expressions in LPS-induced RAW 264.7 cell line.

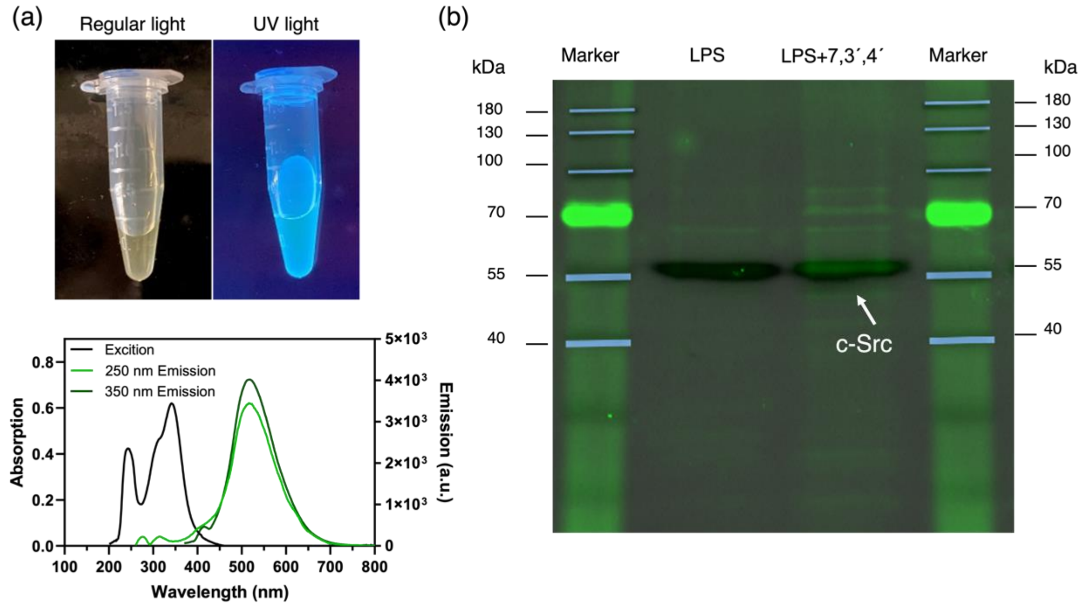

Figure S5 Application of natural fluorescent 7,3´,4´-HOFL for receptor finding. Pictures of 50 μM 7,3´,4´-HOFL under regular (left) and UV light (right), and its fluorescence spectra **(a)**; merged image of fluorescent gel band and c-Src band on Western blotting membrane **(b)**.

Table S1 Sequences of primers for RT-qPCR

Gene	Forward strand	Reverse strand
β-actin	5′-CCACAGCTGAGAGGGAAATC-3′,	5′-AAGGAAGGCTGGAAAAGAGC-3′
IL-1β	5′-GGGCCTCAAAGGAAAGAATC-3′	5′- TACCAGTTGGGGAACTCTGC-3′
IL-6	5′-AGTTGC CTTCTTGGGACTGA-3′	5′-CAGAATGCCATTGCACAAC-3′
TNF-α	5′-AGCCCCCAGTCTGTATCCTT-3′	5′-CATTCGAGGCTCCAGTGAAT-3′
Stat5a	5′-GACGTGGGCTCCTCACACTGA-3′	5′-CGCTGGACTCCATGCTTCTC-3′
Csf2	5′-GAGGATGTGGCTGCAGAATTTAC-3′	5′- CTTCTACCTCTTCATTCAACGTGAC-3′
Il2ra	5′-GGGAAAACGGGGTGGACTC-3′	5′-CTGTGGTGGTTATGGGGCAG-3′
Il13ra2	5′-CCGAAATGTTGATAGCGACAGC-3′	5′-CCAAGCCCTCATACCAGAAAAAC-3′
Ifnb1	5′-GAGCTATTACTGGAGGGTGCAAA-3′	5′-ATCTCTGCTCGGACCACCAT-3′
Mmp13	5′-GGAGCCACAGATGAGCACAGA-3′	5′-TGAACGCTCGCAGTGAAAAG-3′
Mmp3	5′-GCCATCTCTTCCATCCAACA-3′	5′-CCAGGGTGTGAATGCTTTTA-3′
Ccl17	5′-TACCATGAGGTCACTTCAGA TGC-3′	5′-GCACTCTCGGCCTACATTGG-3′
Ccl12	5′-ATTTCCACACTTCTATGCCTCCT-3′	5′-ATCCAGTATGGTCCTGAAGATCA-3′

Table S2 overview of RNA-Sequencing reads

Sample	Raw reads	Raw bases	Clean reads	Clean bases	Q20	Q30	GC_pct
Ctrl_1	93144302	13.97 G	91125112	13.67 G	97.36	92.97	50.09
Ctrl_2	87358694	13.1 G	85473584	12.82 G	97.12	92.52	49.85
Ctrl_3	87548506	13.13 G	85869924	12.88 G	97.17	92.59	49.92
LPS_1	85680904	12.85 G	83528966	12.53 G	97.06	92.43	49.95

LPS_2	87404702	13.11 G	85399376	12.81 G	97.24	92.83	49.98
LPS_3	86720560	13.01 G	84936632	12.74 G	97.08	92.45	50.19
HOFL_634_1	87912690	13.19 G	86092230	12.91 G	97.27	92.82	49.87
HOFL_634_2	86939308	13.04 G	84775824	12.72 G	97.26	92.84	49.79
HOFL_634_3	88468112	13.27 G	86490414	12.97 G	97.27	92.86	50.01
HOFL_734_1	88429030	13.26 G	86421576	12.96 G	97.11	92.49	50.01
HOFL_734_2	88488584	13.27 G	85853778	12.88 G	96.93	92.09	49.85
HOFL_734_3	8836541	13.25 G	85770200	12.87G	97.35	93.05	50.01
