# Supplementary figures and images for: Antioxidant and Anti-Inflammatory Effects of 6,3’,4´- and 7,3´,4´-Trihydroxyflavone on 2D and 3D RAW264.7 Models
Source: Antioxidants (Basel). 2023 Jan 16;12(1):204. doi: 10.3390/antiox12010204 (PMC9855077; doi:10.3390/antiox12010204)

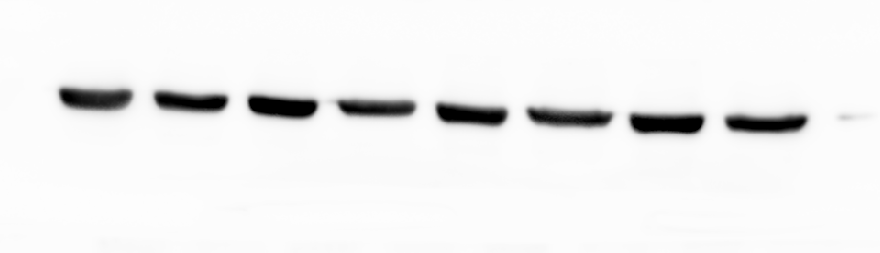

Supplement: Supplementary file 1 [file antioxidants-12-00204-s001.zip › file S1 Western Blots/actin _2.tif]

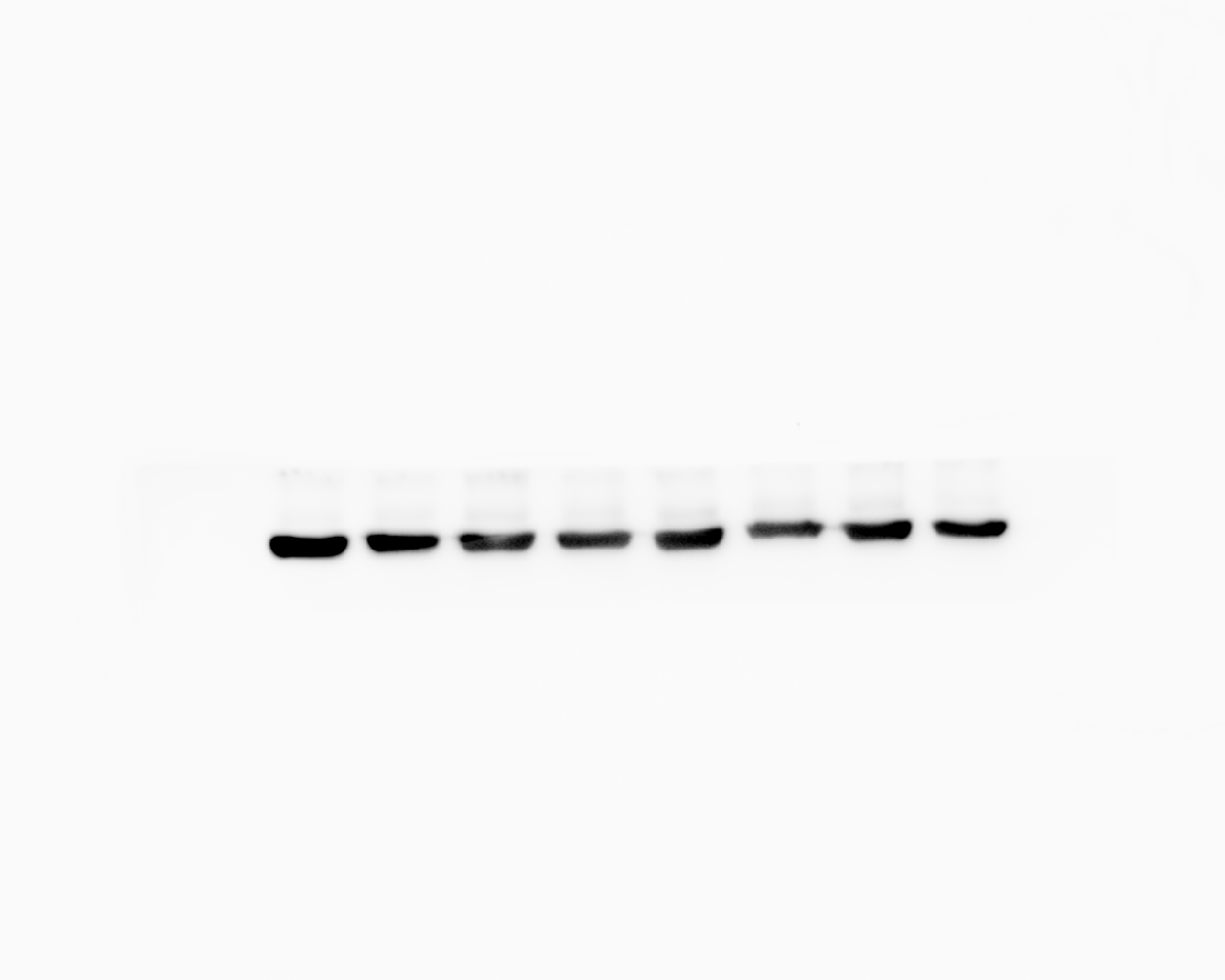

Supplement: Supplementary file 1 [file antioxidants-12-00204-s001.zip › file S1 Western Blots/actin_1.tif]

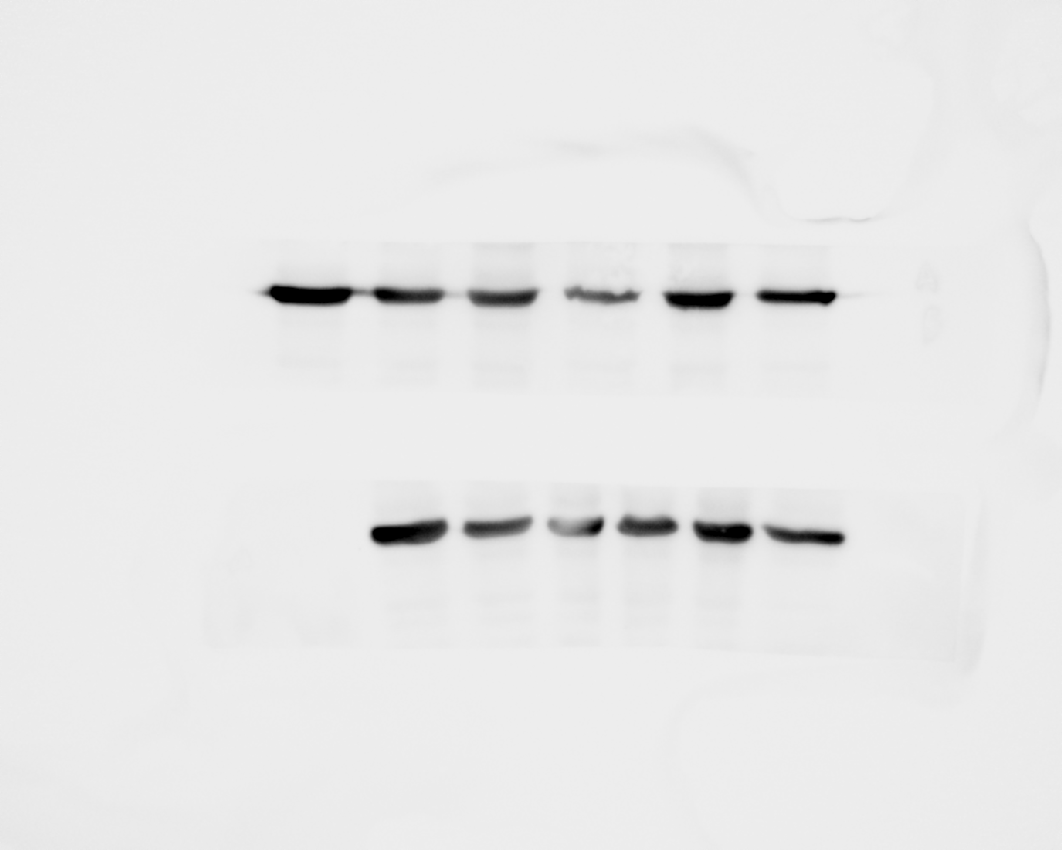

Supplement: Supplementary file 1 [file antioxidants-12-00204-s001.zip › file S1 Western Blots/actin_34.tif]

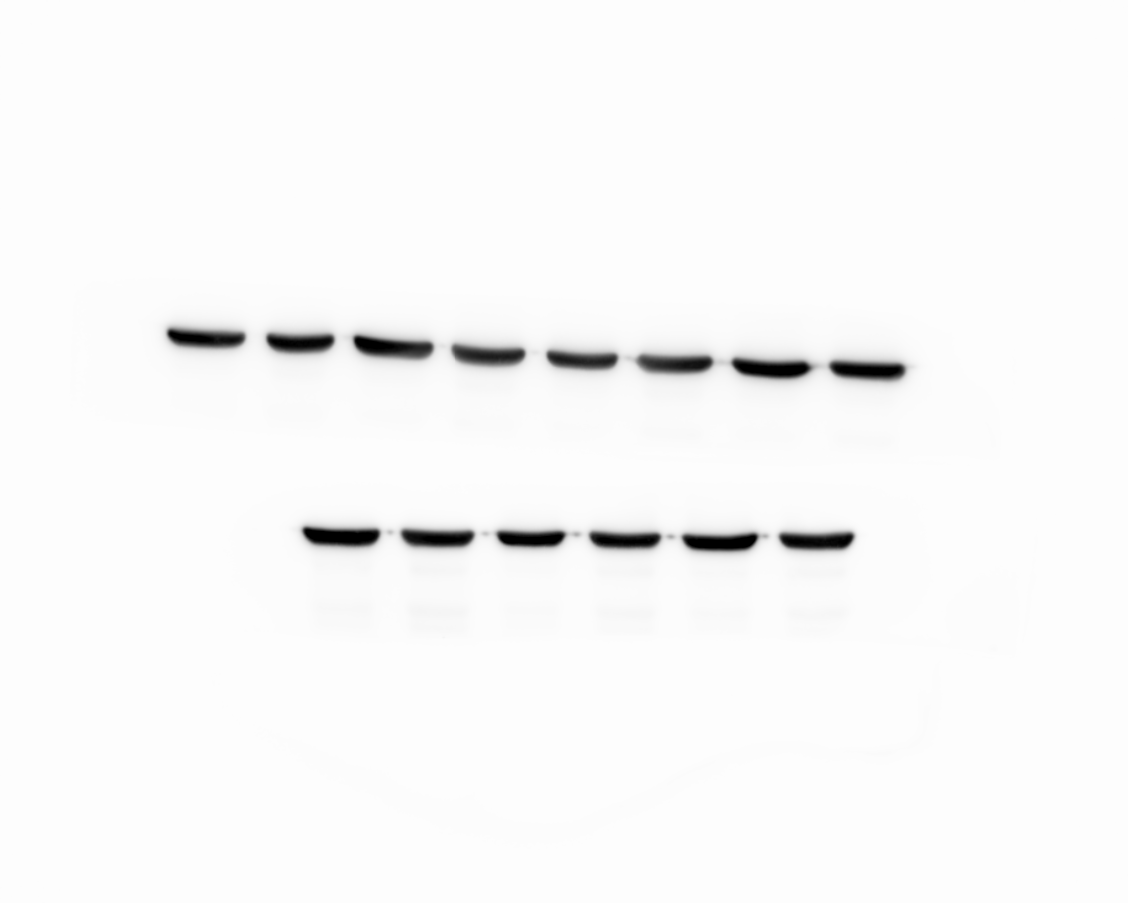

Supplement: Supplementary file 1 [file antioxidants-12-00204-s001.zip › file S1 Western Blots/actin_56 (COX-2).tif]

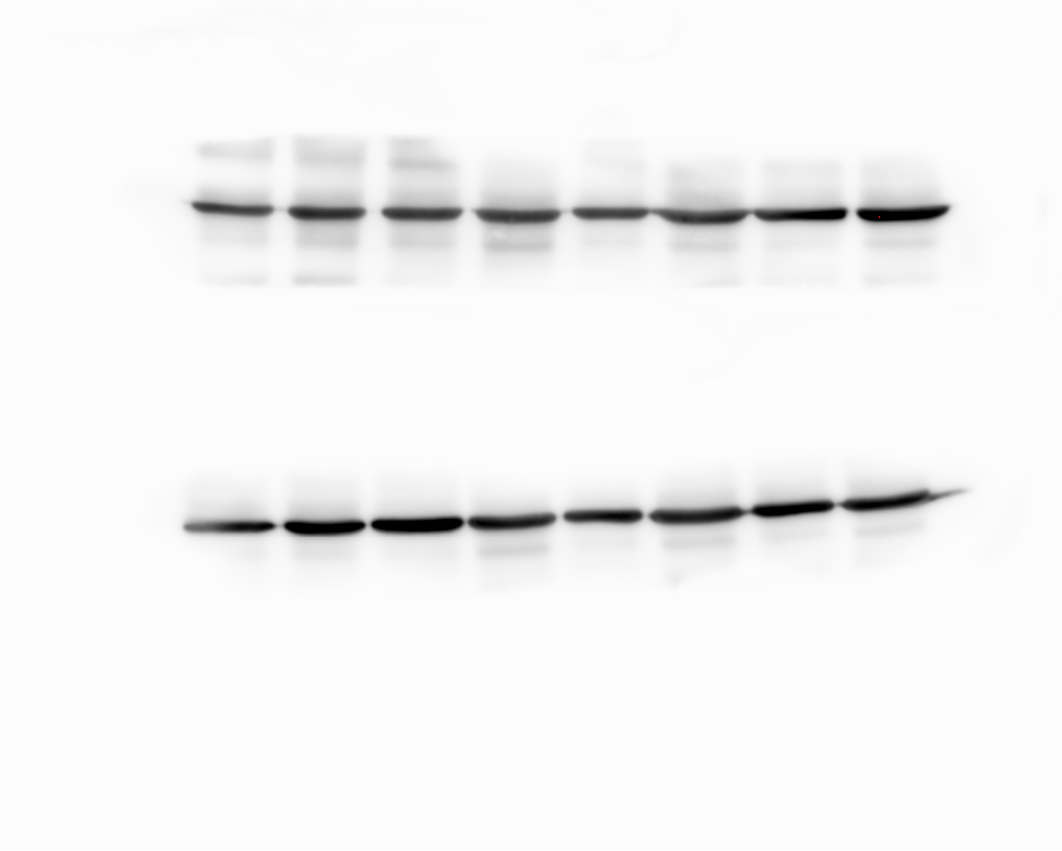

Supplement: Supplementary file 1 [file antioxidants-12-00204-s001.zip › file S1 Western Blots/actin_56 (iNOS).tif]

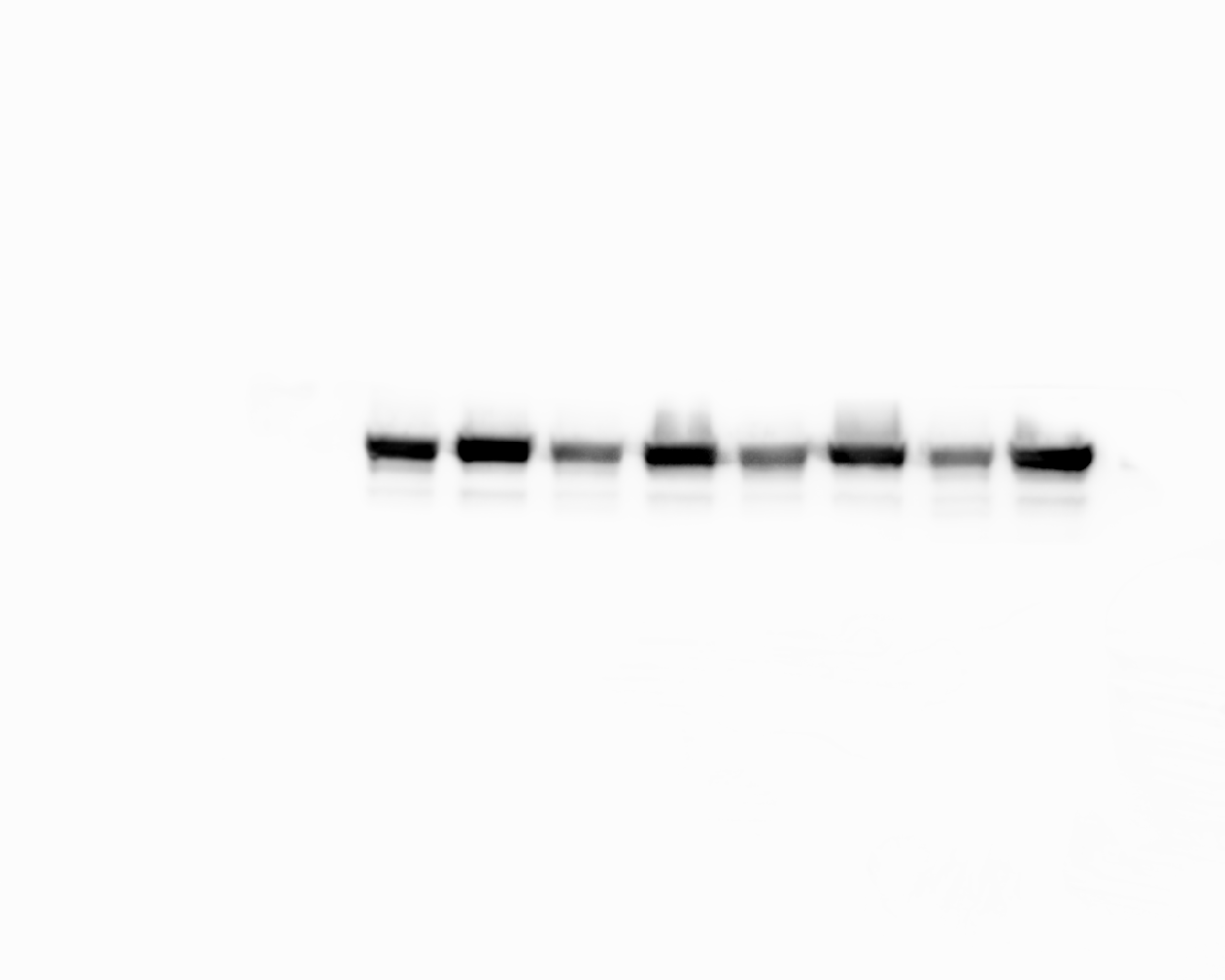

Supplement: Supplementary file 1 [file antioxidants-12-00204-s001.zip › file S1 Western Blots/COX-2_1.tif]

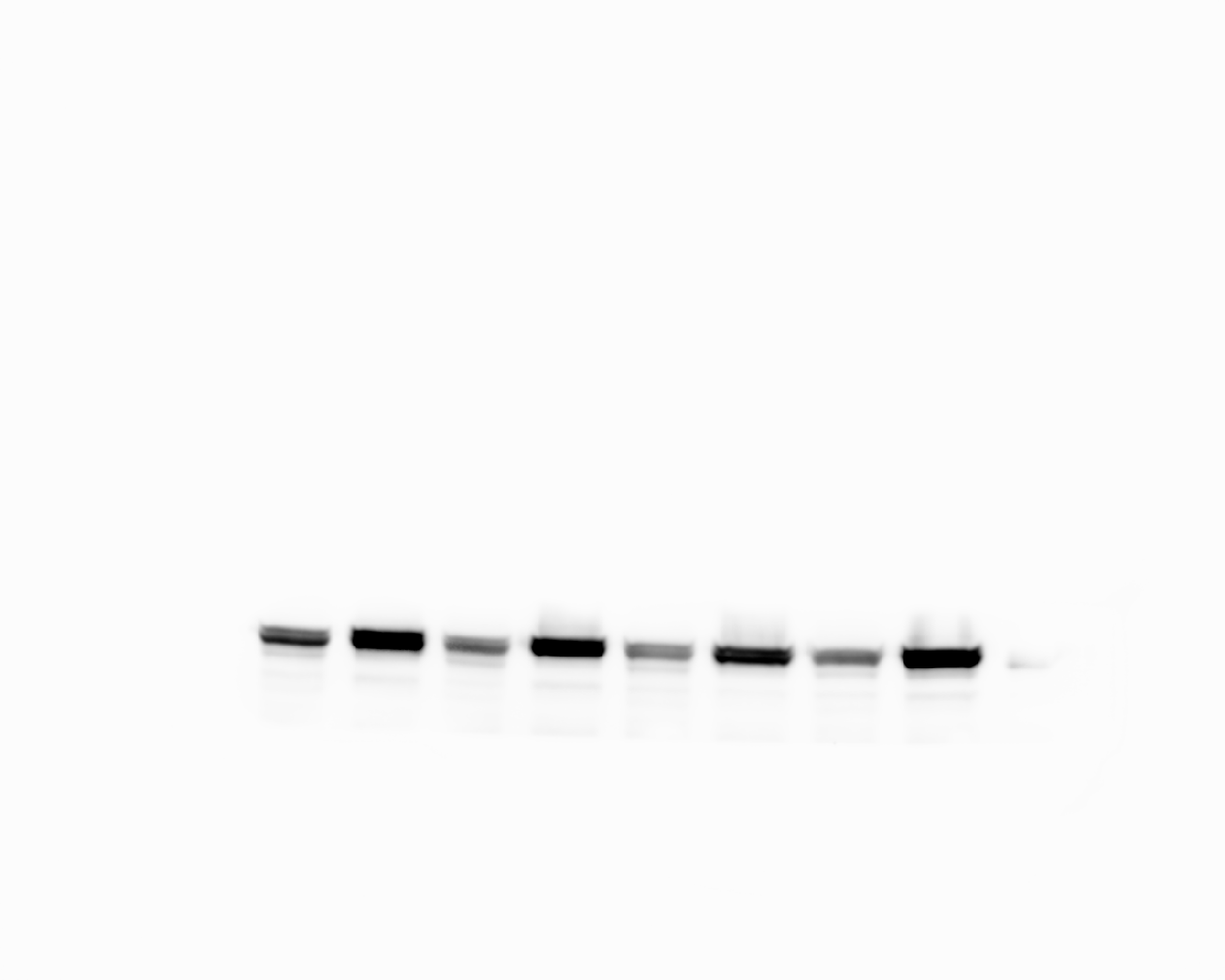

Supplement: Supplementary file 1 [file antioxidants-12-00204-s001.zip › file S1 Western Blots/COX-2_2.tif]

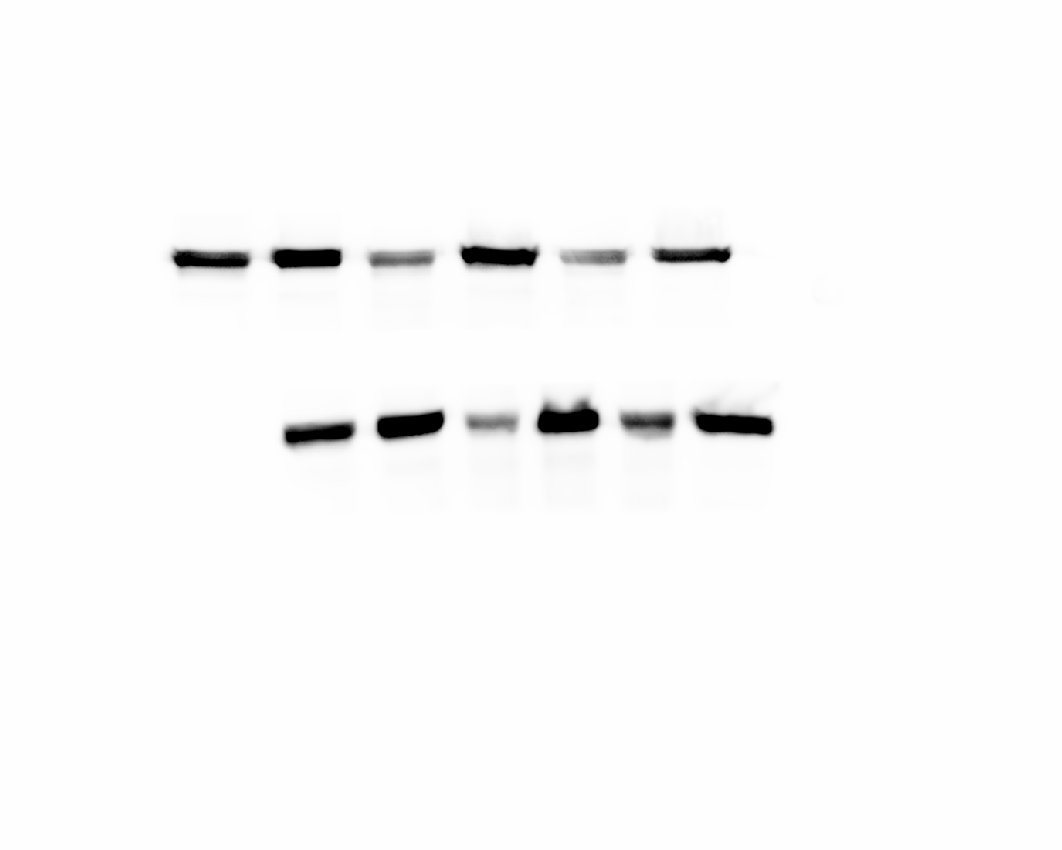

Supplement: Supplementary file 1 [file antioxidants-12-00204-s001.zip › file S1 Western Blots/COX-2_34.tif]

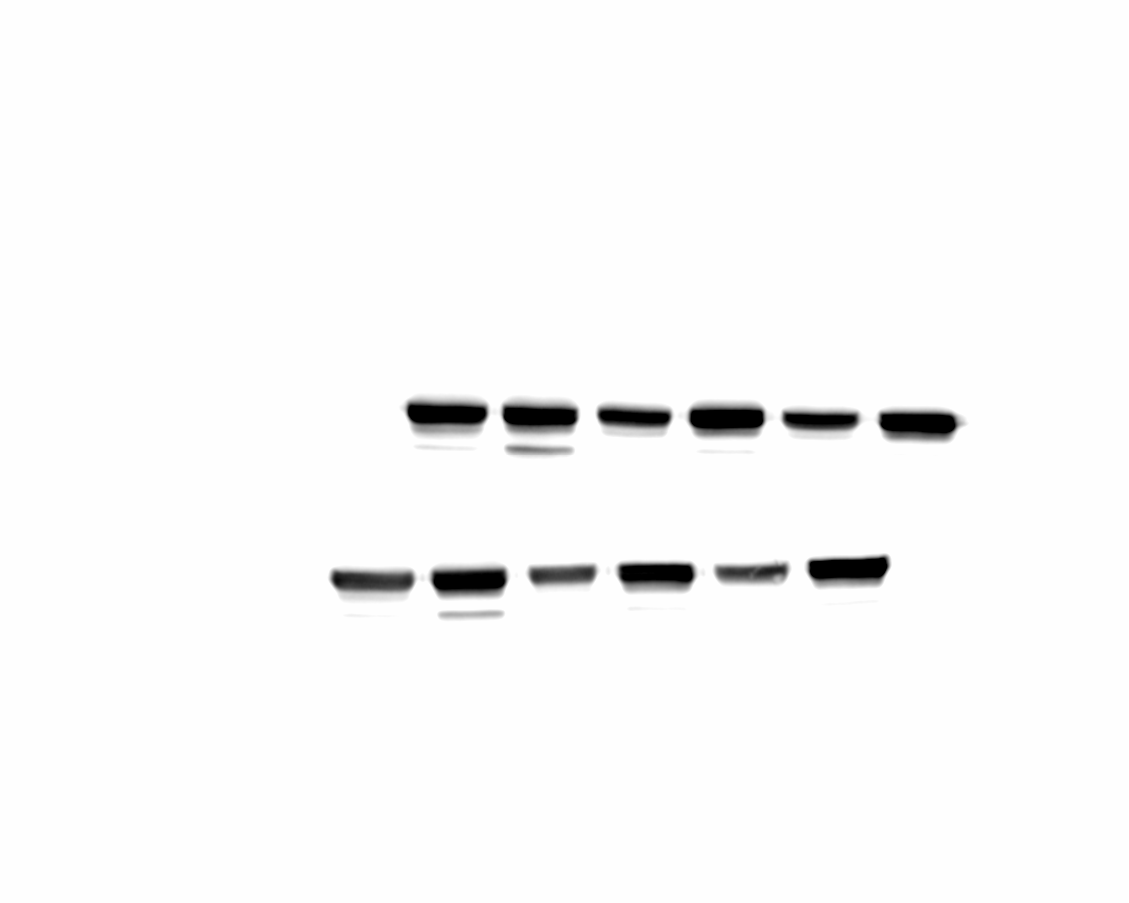

Supplement: Supplementary file 1 [file antioxidants-12-00204-s001.zip › file S1 Western Blots/COX-2_56.tif]

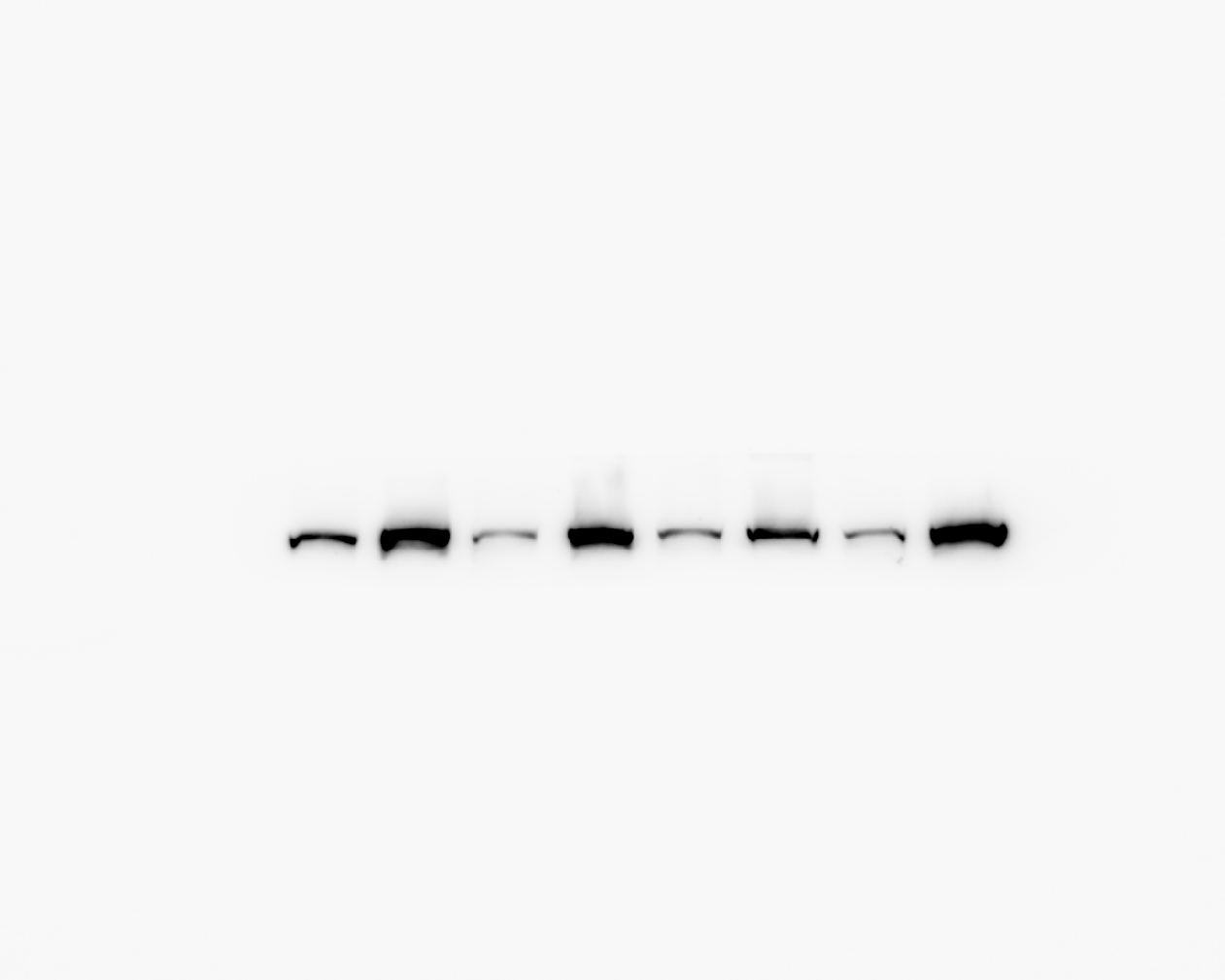

Supplement: Supplementary file 1 [file antioxidants-12-00204-s001.zip › file S1 Western Blots/iNOS_1.tif]

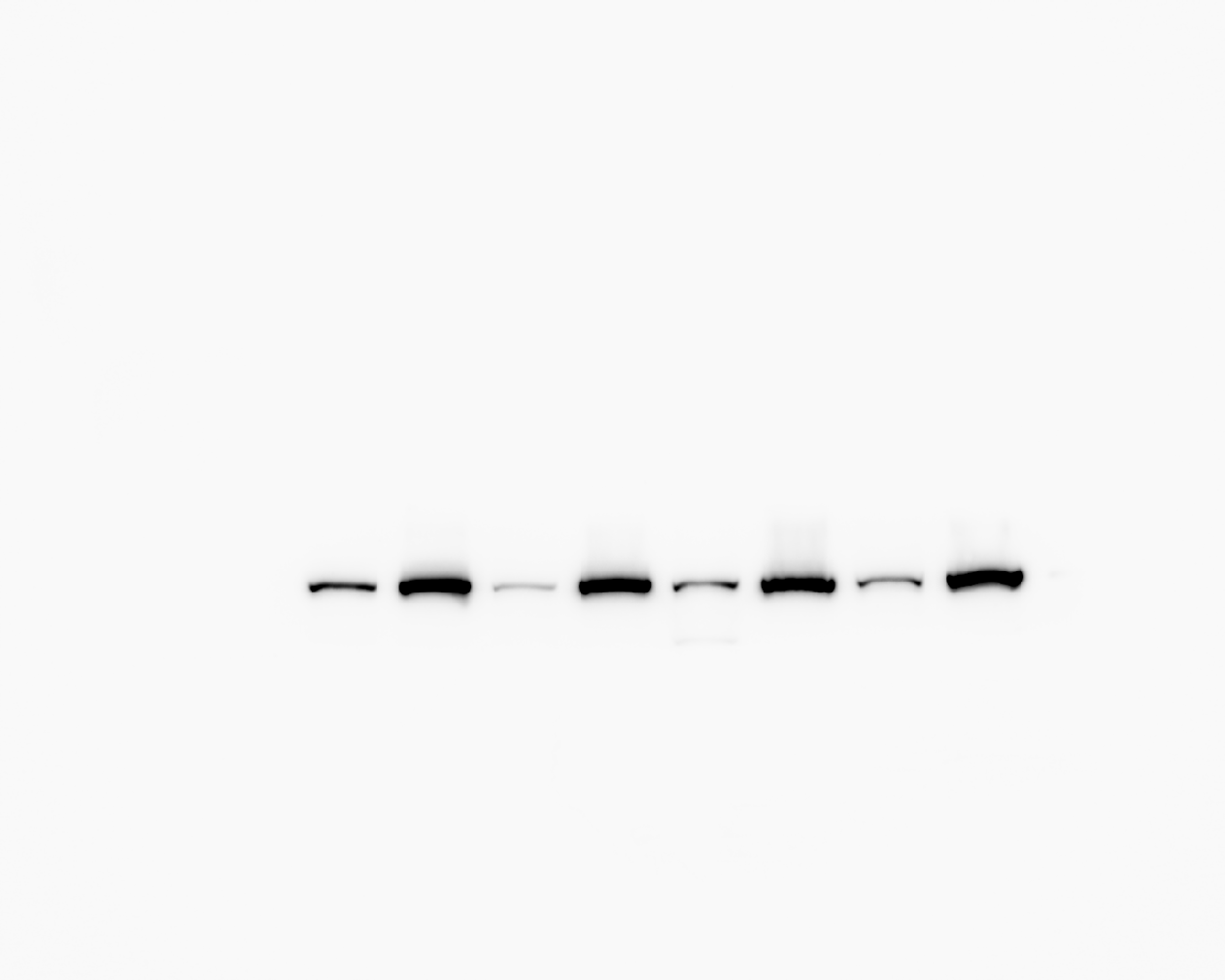

Supplement: Supplementary file 1 [file antioxidants-12-00204-s001.zip › file S1 Western Blots/iNOS_2.tif]

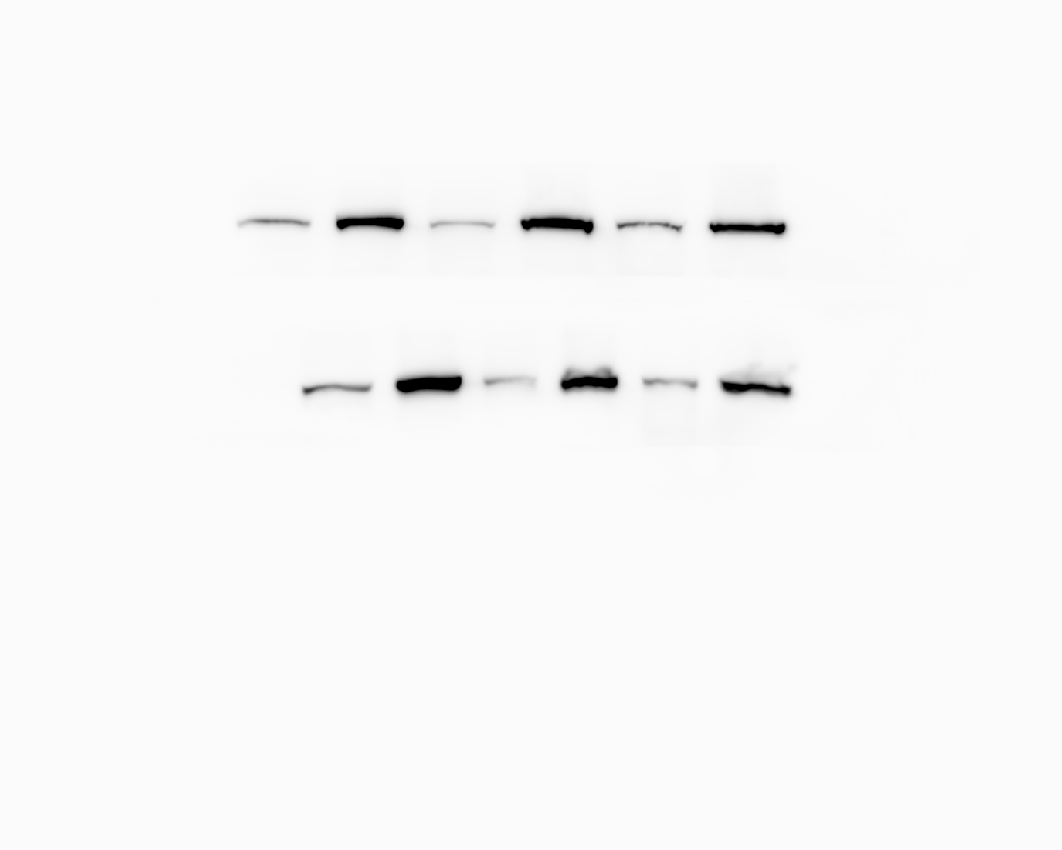

Supplement: Supplementary file 1 [file antioxidants-12-00204-s001.zip › file S1 Western Blots/iNOS_34.tif]

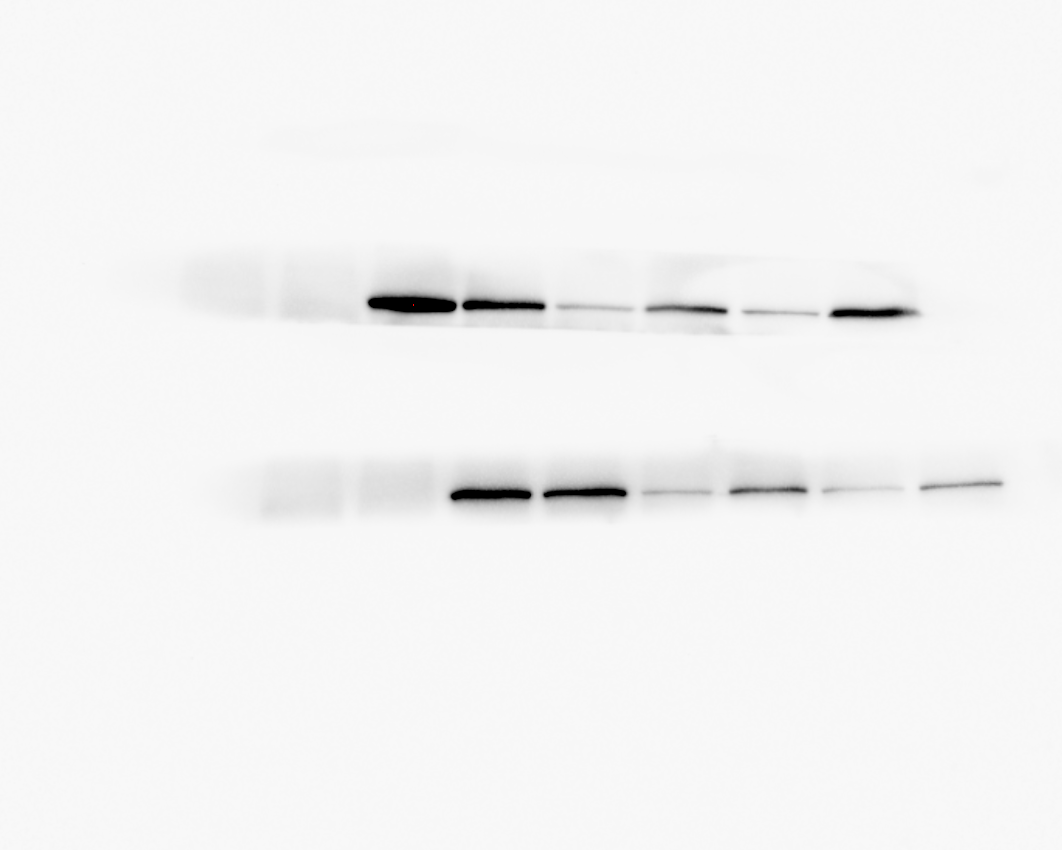

Supplement: Supplementary file 1 [file antioxidants-12-00204-s001.zip › file S1 Western Blots/iNOS_56.tif]
